# Comparative Efficacy of Topical Pyrethroids and Benzoyl Peroxide for Treating Chorioptic Mange in Spanish-Breton Horses

**DOI:** 10.1155/japr/8948099

**Published:** 2025-04-22

**Authors:** Juan D. Carbonell, Nélida Fernández, Manuel J. Escobar, Maria T. Álvarez, Lucia Sánchez, Aday Hernández, Aránzazu Meana

**Affiliations:** ^1^Department of Animal Health, Faculty of Veterinary, Complutense University of Madrid, Madrid, Spain; ^2^Faculty of Veterinary, University Alfonso X El Sabio, Madrid, Spain; ^3^Clinical Horse Veterinarians, Madrid, Spain

**Keywords:** benzoyl peroxide, *Chorioptes bovis*, horse, mange, pyrethroids, topical treatment

## Abstract

Chorioptic mange is a challenging condition to treat due to the superficial locations of the nonhematophagous mite *Chorioptes bovis*, and while topical acaricides are recommended, the clinical feature relapses are frequent. In a double-blinded clinical trial, three randomized groups of Spanish-Breton horses (*n* = 32) naturally infected with *C. bovis* on their legs were evaluated over a period of 70 days. All treatments were applied once every 14 days for three treatments. Before treatment and on Day (D)10, D25, D37, D56, and D70, each leg per animal was scored according to equine pastern dermatitis clinical presentations and severity (1 = mild, 2 = exudative, and 3 = chronic proliferative), and mite reduction was calculated using a 2 *cm* × 6 *cm* adhesive tape counting total mites on D36, D56, and D70. The trial aimed to assess the clinical improvement and efficacy of a topical pyrethroid emulsion alone (Group 1), and in combination with benzoyl peroxide (Group 2), compared to a control group (Group 3). The trial also included environmental disinfection. The acaricide efficacy was determined using Abbott's formula. Our analysis reveals no adverse reactions attributable to the treatment, yet lesions showed limited clinical improvement. Both treatment groups exhibited mite reduction compared to the control. The mite reduction on the evaluated days was 14.58%, 47.62%, and 55.77% for Group 1 and 85.42%, 88.10%, and 78.85% for Group 2, respectively. The mite reduction was significant in Group 2 on D56 and D70 (*p* < 0.0156) compared to the pretreatment and superior to Group 1 (*p* < 0.0229) at the end of the study (D70). The age and mite numbers showed no significant connection. In horses with higher clinical scores, there were no significant changes, most probably due to the short length of the study. The combination of benzoyl peroxide with topical pyrethroids can reduce the numbers of *C. bovis* mites, and it can be considered an alternative therapeutic option in horses.

## 1. Introduction

Horse mange is a contagious, parasitic skin disease characterized by crusting, itching, and alopecia. It is a condition that can be induced by several mite species such as *Sarcoptes scabiei*, *Demodex caballi*, *Psoroptes ovis*, and specially *Chorioptes bovis* (Hering, 1845) [1], a mite that lives and feeds on the epidermal debris that accumulates at the surface of the host's skin [2]. This parasite has a worldwide distribution, and reports came from different continents [3–6]. Mites affect horses of larger breeds [7], and while infection tends to concentrate in the animal's lower extremities [8], it also often affects other body parts where crusts, scales, epidermal collarettes, nodules, excoriations, and ulcers are produced. Moreover, secondary bacterial infection [9, 10] and pruritus often accompany these lesions. Recently, more attention has been paid to *C. bovis* as a possible cause of chronic progressive lymphadenitis (CPL), a condition with high morbidity that affects draft horses [11, 12].

Due to the superficial locations and nonhematophagous behaviour of *C. bovis*, treatment with topical acaricides is recommended [13]. However, the treatment and control of chorioptic mange is challenging [7], and relapses are frequent, most probably due to the difficulties to access the location of the mite in the deeper areas of the pasterns. A variety of active ingredients have been used to treat horses with this disease [14, 15], including macrocyclic lactones [5, 16, 17], selenium sulphide [18], phenylpyrazoles [19], and sulphur [20], all of which are applied to the host. In addition, pyrethroids are used in the environment [5].

Given the varying efficacy of the different treatment options for chorioptic mange in horses, alternative therapeutic approaches should be evaluated that may produce better results in daily practice. While pyrethroids are active against flies, lice, ticks, and mites [21], the use of a keratolytic agent like benzoyl peroxide [22, 23] in conjunction with pyrethroids may facilitate the latter's insecticidal effect on mites.

Pyrethroids modulate sodium channels. They keep sodium channels open, causing hyperexcitability and, in some cases, nerve block. Sodium channels are involved in the propagation of action potentials along nerve axons [24]. This insecticide group acts on the nerve and muscle targets of the mite.

Unlike macrocyclic lactones, pyrethroids do not affect endoparasites, potentially helping prevent anthelmintic resistance and its impact on dung-dwelling insect populations [25, 26].

Benzoyl peroxide acts as a keratolytic agent with antibacterial, anti-inflammatory, exfoliant, and comedolytic properties. It effectively destroys both surface and ductal microorganisms, aided by its lipophilic nature, which allows it to penetrate the pilosebaceous ducts. Upon application to the skin, benzoyl peroxide decomposes to release free oxygen radicals, providing potent bactericidal activity in sebaceous follicles and exerting anti-inflammatory effects. After topical application, benzoyl peroxide penetrates through the stratum corneum and into the follicles, then diffuses into the epidermis and dermis, where it is ultimately converted into benzoic acid [27]. As mentioned, it is a keratolytic agent due to its exfoliating action, which chemically dissolves the bond between dead skin cells and the skin, facilitating their removal.

This study set out to assess the clinical improvement and parasitological efficacy of topical pyrethroid therapy in horses naturally infected with *C. bovis*, used as both a stand-alone treatment or in combination with benzoyl peroxide as an adjuvant.

## 2. Materials and Methods

### 2.1. Horses

Spanish-Breton draft horses (*n* = 32) from a single farm in Madrid (Spain) that had chorioptic mange lesions on their extremities were included in this study. Otherwise, the animals were in good health, with a complete history of vaccinations. The animals were dewormed based on coprology, and they underwent selective parasite control and had never previously been treated for mange. Since these horses were exhibition animals, it was not feasible to clip or shave their limbs.

### 2.2. Inclusion Criteria

All the animals included in this study adhered to the following criteria:

1. Horses exhibiting lesions consistent with mange that were confirmed to host *C. bovis* mites (≥ 1);
2. Horses with lesions consistent with mange but with no definitive confirmation of the presence of *C. bovis* mites;
3. Horses with no lesions but that had been in direct contact or cohabitated with any of the horses that complied with the aforementioned criteria.

The inclusion process involved microscopy examination of adhesive skin tapes for *C. bovis* mites that was performed by a qualified veterinary parasitologist. A second reading, also performed by a veterinary parasitologist, ensured that the mite counts coincided on both occasions. The animal's skin was examined by a clinical horse veterinarian.

The distribution criteria and the assignment of the experimental groups can be found in Table S1. The animals are identified numerically and randomly distributed into the three groups.

### 2.3. Exclusion Criteria

The exclusion criteria applied in this study were as follows:

1. Aggressive animals;
2. Pregnant mares;
3. Horses < 3 years old;
4. Animals for human consumption.

### 2.4. Study Timeline

Each horse was stabled independently in its own facility, in areas designated for their respective study group. The study spanned 70 days, with treatments administered to the horses and disinfection of the environment carried out on Days 1, 21, and 35 considering the biological cycle of the mite, which is completed in 2–3 weeks [28]. Lesions were assessed before each treatment (PRE) and on Days 10, 25, 37, 56, and 70. The number of parasites was also evaluated before the treatments (PRE) and on Days 37, 56, and 70. The study design followed the guidelines for acaricide efficacy in ruminants of the World Association for the Advancement of Veterinary Parasitology (WAAVP) [29]. As there are no specific guidelines available to evaluate acaricide efficacy in equids, the guidelines on specific efficacy requirements for ectoparasiticides in cattle laid down by the European Medicines Agency (EMA) and the Committee for Medicinal Products for Veterinary Use [30] were followed.

### 2.5. Lesion Assessment

The lesions on each of the extremities were classified based on the clinical presentation of pastern dermatitis, scored as 1 (mild), 2 (exudative), or 3 (proliferative) [31]. The scores for all legs were combined to give an overall score for each horse, ranging from 1 to 12. Any relevant information and clinical signs were recorded on a clinical, dermatological record sheet [32].

### 2.6. Mite Count

Mite counts were obtained using a 2 *cm* × 6 *cm* (12 cm^2^ area) adhesive strip [33]. The same mange-type lesions were selected on each leg (pastern) to count mites over a total surface area of 48 cm^2^ per animal, where *C. bovis* tends to localize [34]. The tape was pressed onto the centre, above, below, and on both sides of the lesion. Subsequently, this tape was examined under a microscope by two veterinarians, each one on a different day. Importantly, all the veterinarians are blind to the group each horse belonged to.

### 2.7. Protocol for Topical Treatment

This was a double-blind, randomized study. Horses meeting the inclusion criteria were assigned to one of the three groups, with each of the groups containing an equivalent initial parasitic burden and horses of the same age. The two treatment groups and the control group received their corresponding applications on Days 1, 21, and 35.

Group 1 (*n* = 9): These animals received ARPON Diazipol off label (tetramethrin 0.09%, cypermethrin 8.50%, and deltamethrin 1.25%) (Zotal, Spain), applying a concentrated emulsion (100 mg/mL) cutaneously to the horses. The product was applied according to the manufacturer's technical data sheet, preparing the spray emulsion by diluting 100 mL of the medication in 900 mL of water. The product was administered to each leg using a pressurized spray canister (500 mL/horse, 125 mL/leg), applied against the direction of hair growth and at a distance of 10 cm to ensure thorough skin saturation. The diluted emulsion applied to the horses' skin was equivalent to 10 mg/mL.

Group 2 (*n* = 11): These animals initially received Benzac Wash gel, Galderma (50 mg/g benzoyl peroxide). An initial application of Benzac was applied and left to act for 15 min before being washed with water. Subsequently, the same treatment regime for Group 1 animals was followed.

Group 3 (*n* = 12): The animals in the control group were sprayed with water following a similar protocol to Group 1, yet without any of the active ingredients of interest in the study.

In addition, Zotal Z (0.1% glycolic acid, 4% biphenyl-2-ol, 0.9% chlorocresol, aromatic hydrocarbons c9–c12, and benzene distillation) (Zotal, Spain) was used as a disinfectant for the animal boxes and common areas used by the horses. The disinfectant was applied according to the manufacturer's instructions, diluting the product to 5% in water and using a pressurized spray canister. The product was sprayed onto the floor and walls on Days 1, 21, and 35 until they were wet. Organic matter and bedding were removed daily throughout the study.

### 2.8. Statistics and Efficacy

The statistical analysis was performed with the SAS software package (Version 9.4: SAS Institute Inc., Cary, North Carolina, United States). The mite reduction in each treatment group was analysed using the Wilcoxon signed-rank test, whereas the reduction of mites between treatment groups was analysed using the Wilcoxon rank-sum test [35]. Furthermore, this study analysed the relationships between the age of the horses and the clinical score of the lesions or the parasite burden using Spearman's correlation. Values were considered significantly different at *p* < 0.05. The acaricide efficacy of both treatments was determined using Abbott's formula [%efficacy = (*C* − *T*)/*C* × 100] [36, 37], comparing the reduction in mite number in the treatment groups with the control group after the last treatment. In this formula, *C* and *T* are the geometric mite counts in the control and treated groups, respectively. A treatment is considered effective when there is a reduction of ≥ 90% (WAAVP: [29]).

## 3. Results

**Figure 1:**
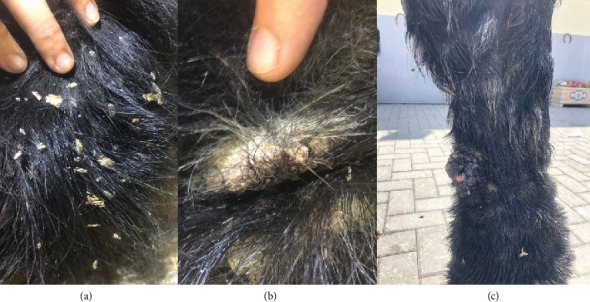
Lesions from (a) scales, (b) crust, and (c) nodules on a horse's extremities.

The median age of the animals was 11 years old (range, 3–22 years), with 23 Spanish-Breton horses meeting the first inclusion criteria (confirmed *C. bovis* mites and lesions), nine meeting the second criteria (lesions resembling mange but unconfirmed mite presence), and none meeting the third criteria (horses without lesions that had direct contact or cohabitated with those meeting Criteria 1 and 2). The horses presented various lesions and different levels of pruritus, consistent with the three clinical forms of pastern dermatitis and chorioptic mange. The most prevalent lesions were crusts, scaling, leuchotrichia, and nodules (Figure 1), and the clinical scores before, during, and after treatment were summarized here (Table 1).

During the study, no significant clinical improvement was observed in any of the groups, although a direct correlation was evident between the severity of clinical signs and the age of the horses in the study. The clinical scores were higher in older horses at the outset of the trial (*p* = 0.0006) and at its end (*p* = 0.0003). Nevertheless, there was no significant correlation between the age and the number of mites at these specific time points (*p* = 0.4941 and *p* = 0.1048, respectively).

**Figure 2:**
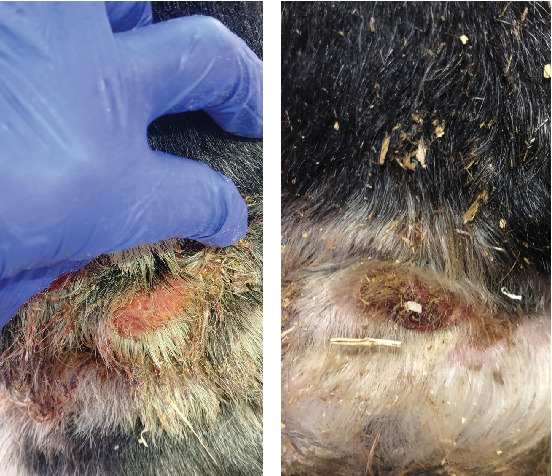
Chorioptic mange lesion before (a) and during treatment (b) with pyrethroids and 5% benzoyl peroxide. Note the decrease in the crusts and the healing of an active lesion.

**Figure 3:**
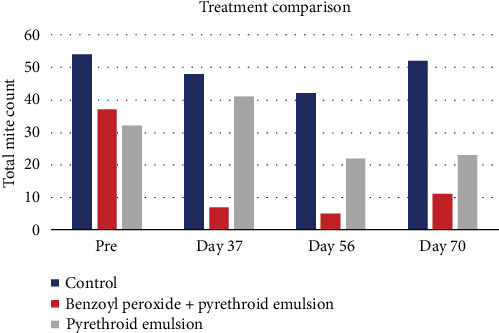
Comparison of the effect of the treatments on the number of mites. Variation in the number of *C. bovis* mites recovered on an adhesive strip on different days.

The treatments were well tolerated by the horses, and no adverse reactions were observed (Figure 2). The *C. bovis* mite counts in naturally infected horses coincided between the two veterinarians (Table 2), and fewer mites were detected in both treatment groups compared to the controls (Figure 3). The efficacy of the pyrethroid emulsion (ARPON Diazipol, Zotal), alone (Group 1), and in combination with benzoyl peroxide gel (Group 2, 50 mg/g, Benzac Wash, Galderma) was assessed on Days 37, 56, and 70. The efficacy on each day of evaluation was 14.58%, 47.62%, and 55.77% for Group 1 and 85.42%, 88.10%, and 78.85% for Group 2, respectively. This reflects a significant reduction in Group 2 on Days 56 and 70 (*p* = 0.0156) compared to the pretreatment stage and superior to Group 1 at the end of the study (*p* = 0.0229).

## 4. Discussion

Chorioptic mange is a common dermatitis for which no medications have been licensed in Spain, nor in many other countries [14]. Moreover, to our knowledge, there are no ongoing studies into the efficacy of topical pyrethroids for the treatment of *C. bovis* in equids, although it is a treatment that has been used for environmental control of the mite in facilities with horses [5] and in vitro, against representative species of biting midge (*Culicoides nubeculosus*) and mosquitoes (*Aedes aegypti* and *Culex quinquefasciatus*) that affect horses [38]. There is evidence that this approach could be beneficial to treat this condition in equids [39], which led us to carry out this first double-blind, randomized, prospective study comparing two different treatment protocols on Spanish-Breton horses naturally infected with *C. bovis*. Unlike macrocyclic lactones, pyrethroids have no impact on the endoparasites of horses, which could be advantageous in preventing anthelmintic resistance [25] and its apparent effect on the dung-dwelling entomofauna populations [26]. In this study, a pyrethroid emulsion containing tetramethrin (0.09%), cypermethrin (8.50%), and deltamethrin (1.25%) was used, which reduced the total number of mites in the two treatment groups throughout the study compared to the controls, the latter maintaining a fairly constant number of mites. This formulation was applied independently and in conjunction with benzoyl peroxide, even though the manufacturer does not indicate its suitability to treat chorioptic mange in horses.

The study was designed in accordance with the recommendations of WAAVP [29] and EMA to evaluate ectoparasiticide use in ruminants [30]. As such, an adequate sample size of animals (*n* = 32) was assigned to each treatment group, with more than six animals, and a control group was included that exceeded the minimum 25% of the animals tested, as also suggested for field studies [29]. Unfortunately, despite including a substantial number of horses, it was not possible to carry out a multicentre field study encompassing different geographical regions at this time. Standardized protocols exist to study anthelmintic efficacy in horses [40], as well as guidelines to control external parasites and vectors [41, 42]. However, unlike other species, there are still no specific guidelines to evaluate the efficacy of ectoparasiticides in equids [29]. Given the evidence of mange in horses [5, 43, 44], developing these guidelines would help standardize efficacy studies such as these.

No clinical improvement was evident in our study, and the clinical scores remained the same throughout the study, in contrast to other studies that reported an improvement in pruritus and specific lesions of chorioptic mange, or of CPL [16]. Elsewhere, data similar to ours was reported concerning the relationship between the age and the severity of skin lesions [7]. The clinical score of older horses was significantly higher at the beginning and end of the study, although there was no significant correlation between the age and the number of mites at these time points. This result may be due to differences in the clinical assessment of pastern dermatitis [31], as opposed to the parameters that are specific to others conditions like CPL [11, 12]. It has been proposed that chronic inflammation induced by agents such as *C. bovis* could cause CPL in draft horses [11]. Thus, the lesions witnessed in some of the animals studied here could be due to CPL in conjunction with the presence of *C. bovis*. We clinically suspected that some horses in our study had CPL, based on the nature of the signs and lesions, which were associated with the horse's age and breed. The histology and diagnostic imaging of the affected areas would have been necessary for a more definitive diagnosis [11], and the lack of these diagnostic methods could be a limiting factor in the present study. Distinct results were obtained in similar prospective studies where a significant reduction in mite number and pruritus was observed but not that of the skin lesions [45]. By contrast, an alternative study did not demonstrate a significant reduction in mites or a general improvement in the skin condition, with skin folds and pruritus persisting, although a marked specific improvement in the crust grades was produced by the treatment [7]. These changes would reflect an amelioration in mange-associated lesions but not that of other types of skin lesions, such as CPL, the mode of action of the treatment, season of the year, or management that is not discussed. Elsewhere, the persistence of skin folds was accompanied by a significant improvement in pruritus and skin lesions associated with both chorioptic mange and CPL [16]. We consider that it will be important to perform a prospective long-term study to determine whether controlling chorioptic mange in horses from an early age could prevent the appearance and development of CPL.

As mentioned previously [7], the horse's caretakers were often opposed to clipping in conjunction with treatment so as to maintain the aesthetic appearance of the horses, despite this recommendation when applying topical applications. When applied to unclipped fetlocks, the efficacy of treatment may be affected, yet it is a common practice in daily clinical practice to leave them unclipped due to animal management or caretaker preference. Nevertheless, for animal welfare and to treat them properly, it is recommendable to ensure that the applications are left for enough time for the product to come into contact with the skin, as ensured here.

We observed a general trend towards a reduction in the number of mites in the groups receiving the treatments, with greater efficacy exhibited by Group 2 than Group 1, which probably reflects the beneficial effect of benzoyl peroxide's keratolytic and antibacterial actions [20]. These properties will assist pyrethroid penetration by eliminating the crusts and scaling. However, the reduction in the number of mites should be verified by studying a control group treated only with benzoyl peroxide. Just as its chemical action allows for greater exposure of the mite to the paralytic effect of the pyrethroid, the mechanical application of the product can similarly aid in reducing mites.

The time interval of topical application was similar to that used elsewhere [7], yet much longer than in other studies [16, 20]. Some other studies failed to include control groups [18, 45], which means that the relative efficacy achieved cannot be compared with those studies that did, given that the calculations are based on the presence or absence of mites in each individual and not on the collective number of mites per group. It is worth noting that a reduction in mite numbers was produced in the control groups in some studies that included environmental control [7]. Alternatively, when psoroptic mange was treated with eprinomectin, the control group recorded a 30% reduction at the end of the trial [46]. This suggests that there could be an influence of general environmental control that can synergise with the topical treatment of some test groups, evident through an impact on the mite numbers in the control horses. Therefore, to control chorioptic mange, it is essential to include steps aimed at environmental management at the facilities in order to enhance treatment performance in each individual horse, reducing the presence of the contagion given that *C. bovis* can survive for extended periods outside the host [18]. In this study, bedding was removed daily, and a disinfectant was applied. The reduction in mite populations could be beneficial, as it may be related to the host's inflammatory response and the pathophysiology of other conditions affecting the skin of the pastern, whose mechanisms of action are still unknown. Last but not least, it would be interesting to study the acaricidal effects of natural compounds on mites affecting horses [47].

In conclusion, the combination of benzoyl peroxide with topical pyrethroids can reduce the numbers of *C. bovis* mites in these horses yet did not significantly improve clinical scores and can be considered an alternative therapeutic option in horses, especially those without CPL lesions.

## Figures and Tables

**Table 1:** Clinical scores. The clinical scores obtained for the individual legs of each horse before, during, and after treatment. The values in bold correspond to the horses from inclusion Criteria 2.

**Clinical score**								
**Horse ID**	**Age (years)**	**Group**	**PRE**	**Day 10**	**Day 25**	**Day 37**	**Day 56**	**Day 70**
1	18	1	12	12	12	12	12	12
9	9	1	8	4	4	5	4	6
11	18	1	12	12	12	12	12	12
15	17	1	12	12	12	12	12	12
16	3	1	6	4	4	4	4	4
20	17	1	12	12	12	12	12	12
23	3	1	6	4	4	4	4	4
**28**	**6**	**1**	**4**	**4**	**4**	**4**	**4**	**8**
**31**	**18**	**1**	**4**	**4**	**4**	**4**	**4**	**4**

4	5	2	4	4	4	4	4	4
5	8	2	12	12	12	12	12	12
8	7	2	8	4	4	4	4	12
10	9	2	12	12	12	12	12	12
14	13	2	4	4	4	4	4	4
17	20	2	8	8	8	8	8	8
19	4	2	4	4	4	4	4	4
22	10	2	10	10	12	12	8	12
**29**	**6**	**2**	**4**	**4**	**4**	**4**	**4**	**4**
**30**	**22**	**2**	**12**	**12**	**12**	**12**	**12**	**12**
**32**	**11**	**2**	**12**	**12**	**12**	**12**	**12**	**12**

2	17	3	12	12	12	12	12	12
3	14	3	8	8	12	12	12	12
6	20	3	12	12	12	12	12	12
7	5	3	8	4	4	5	4	4
12	5	3	6	4	4	5	6	4
13	16	3	5	5	4	8	5	4
18	13	3	12	12	12	12	12	12
21	5	3	4	4	4	8	4	4
**24**	**9**	**3**	**4**	**4**	**4**	**4**	**4**	**4**
**25**	**13**	**3**	**8**	**8**	**8**	**8**	**8**	**8**
**26**	**6**	**3**	**4**	**4**	**4**	**4**	**8**	**8**
**27**	**5**	**3**	**4**	**4**	**4**	**4**	**4**	**4**

*Note:* PRE, pretreatment.

**Table 2:** The number of *C. bovis* mites recovered from lesions with an adhesive strip on each of the four legs of naturally infected horses, before, during, and after treatment. The values in bold correspond to the horses from inclusion Criteria 2.

**Mite reduction**						
**Horse ID**	**Age (years)**	**Group**	**Pretreatment**	**Day 37**	**Day 56**	**Day 70**
1	18	1	18	21	8	10
9	9	1	6	0	0	4
11	18	1	3	14	5	2
15	17	1	2	2	5	3
16	3	1	1	0	0	0
20	17	1	1	0	2	2
23	3	1	1	0	0	1
**28**	**6**	**1**	**0**	**4**	**2**	**0**
**31**	**18**	**1**	**0**	**0**	**0**	**1**
Total		1	32	41	22	23

4	5	2	13	0	0	7
5	8	2	9	0	2	0
8	7	2	7	0	0	1
10	9	2	3	2	1	3
14	13	2	2	1	0	0
17	20	2	1	1	1	0
19	4	2	1	0	0	0
22	10	2	1	2	0	0
**29**	**6**	**2**	**0**	**0**	**0**	**0**
**30**	**22**	**2**	**0**	**0**	**1**	**0**
**32**	**11**	**2**	**0**	**1**	**0**	**0**
Total		2	37	7	5	11

2	17	3	16	3	0	6
3	14	3	15	25	29	29
6	20	3	9	14	9	8
7	5	3	7	4	0	4
12	5	3	3	0	0	0
13	16	3	2	0	0	0
18	13	3	1	0	2	3
21	5	3	1	1	1	0
**24**	**9**	**3**	**0**	**1**	**0**	**2**
**25**	**13**	**3**	**0**	**0**	**0**	**0**
**26**	**6**	**3**	**0**	**0**	**1**	**0**
**27**	**5**	**3**	**0**	**0**	**0**	**0**
Total		3	54	48	42	52

## Data Availability

The datasets generated and analysed during the current study are not publicly available but can be obtained from the corresponding author upon reasonable request. However, the supporting information and tables included in this article correspond to the data used in this study.

## Supporting Information

Supporting Information Additional supporting information can be found online in the Supporting Information section. The following describes the criteria for horse selection, identification, and the method used for their random assignment into experimental groups.
